# Evaluating seroprevalence to circumsporozoite protein to estimate exposure to three species of *Plasmodium* in the Brazilian Amazon

**DOI:** 10.1186/s40249-018-0428-1

**Published:** 2018-05-14

**Authors:** Virginia Araujo Pereira, Juan Camilo Sánchez-Arcila, Mariana Pinheiro Alves Vasconcelos, Amanda Ribeiro Ferreira, Lorene de Souza Videira, Antonio Teva, Daiana Perce-da-Silva, Maria Teresa Queiroz Marques, Luzia Helena de Carvalho, Dalma Maria Banic, Luiz Cristóvão Sobrino Pôrto, Joseli Oliveira-Ferreira

**Affiliations:** 1 Laboratory of Immunoparasitology, Oswaldo Cruz Institute/Fiocruz, Av. Brasil 4365, Manguinhos, Rio de Janeiro, Brazil; 2 Institute of Infectology Emilio Ribas, São Paulo, São Paulo Brazil; 3 Laboratory of Immunodiagnosis, Departament of Biological Sciences, National School of Public Health/Fiocruz, Rio de Janeiro, Brazil; 4 Laboratory of Clinical Immunology, Oswaldo Cruz Institute/Fiocruz, Rio de Janeiro, Brazil; 5 Laboratory of Histocompatibility and Cryopreservation – HLA of DHE/IBRAG/UERJ, Rio de Janeiro, Brazil; 6 Molecular Biology and Malaria Immunology Research Group, Centro de Pesquisas René Rachou/Fiocruz, Belo Horizonte, Minas Gerais Brazil

**Keywords:** Malaria, Circumsporozoite protein, Serological marker, Human leucocyte antigen, IgG antibody, Porto Velho, Rondônia, Brazil

## Abstract

**Background:**

Brazil has seen a great decline in malaria and the country is moving towards elimination. However, for eventual elimination, the control program needs efficient tools in order to monitor malaria exposure and transmission. In this study, we aimed to evaluate whether seroprevalence to the circumsporozoite protein (CSP) is a good tool for monitoring the exposure to and/or evaluating the burden and distribution of *Plasmodium* species in the Brazilian Amazon.

**Methods:**

Cross-sectional surveys were conducted in a rural area of Porto Velho, Rondônia state. Parasite infection was detected by microscopy and polymerase chain reaction. Antibodies to the sporozoite CSP repeats of *Plasmodium vivax*, *P. falciparum*, and *P. malariae* (PvCS, PfCS, and PmCS) were detected using the enzyme-linked immunosorbent assay technique. Human leukocyte antigen (HLA)-DRB1 and DQB1 genes were typed using Luminex® xMAP® technology.

**Results:**

The prevalence of immunoglobulin G against *P. vivax* CSP peptide (62%) was higher than *P. falciparum* (49%) and *P. malariae* (46%) CSP peptide. Most of the studied individuals had antibodies to at least one of the three peptides (72%)**,** 34% had antibodies to all three peptides and 28% were non-responders. Although the majority of the population was not infected at the time of the survey, 74.3% of parasite-negative individuals had antibodies to at least one of the CSPs. Importantly, among individuals carrying the haplotypes DRB1*04~DQB1*03, there was a significantly higher frequency of PfCS responders, and DRB1*16~DQB1*03 haplotype for PvCS and PfCS responders. In contrast, HLA-DRB1*01 and HLA-DQB1*05 allelic groups were associated with a lack of antibodies to *P. vivax* and *P. falciparum* CSP repeats, and the haplotype DRB1*01~DQB1*05 was also associated with non-responders, including non-responders to *P. malariae*.

**Conclusions:**

Our results show that in low transmission settings, naturally acquired antibody responses against the CSP repeats of *P. vivax*, *P. falciparum*, and *P. malariae* in a single cross-sectional study may not represent a valuable marker for monitoring recent malaria exposure, especially in an area with a high prevalence of *P. vivax*. Furthermore, HLA class II molecules play an important role in antibody response and require further study with a larger sample size. It will be of interest to consider HLA analysis when using serosurveillance to monitor malaria exposure among genetically diverse populations.

**Electronic supplementary material:**

The online version of this article (10.1186/s40249-018-0428-1) contains supplementary material, which is available to authorized users.

## Multilingual abstracts

Please see Additional file 1 for translations of the abstract into the five official working languages of the United Nations.

## Background

Malaria remains an acute public health problem, particularly in Sub-Saharan Africa, despite an impressive reduction in the number of cases globally. According to the latest estimates from the World Health Organization, there were 212 million new cases of malaria and 429 000 deaths in 2015 worldwide [1].

In Brazil, major successful control efforts have contributed to the number of cases declining from 615 246 in 2000 to 143 145 in 2014, a reduction of 76.7% [2]. Based on this achievement, the Ministry of Health launched the Plan for the Elimination of Malaria in Brazil, in November 2015 [3]. In 2016, 128 503 cases were reported in Brazil, with *Plasmodium vivax* representing 88.3%, *P. falciparum* 11.7%, and only a few cases of *P. malariae* being reported (however, molecular techniques have detected this species in 9–12% of malaria patients in selected settings) [4, 5]. While the prospects for eliminating *P. falciparum* malaria are good, a progressive increase in *P. vivax* cases remains a challenge [6, 7]. In *P. vivax* infections, the presence of asymptomatic carriers and the possibility of relapse impose additional challenges for surveillance. In addition, there is no current method for diagnosing hypnozoite carriers and *P. vivax* infections that recur after drug treatment can be attributed to relapse arising from the dormant liver stages (hypnozoites) or recrudescence originating from asexual blood-stage parasites that survived drug treatment, or a reinfection resulting from a new mosquito inoculation [8].

The challenge to sustain and ensure malaria elimination is to detect asymptomatic infections in order to block transmission. In endemic areas, the risk of infection has been routinely estimated by parasite prevalence using microscopy and rapid diagnostic tests (RDTs) or estimated using the entomological inoculation rate (EIR) [9, 10]. Microscopy and RDT are easy to deploy, but are not sensitive enough to detect low-grade infections that are typical of settings with residual malaria transmission. The EIR method is time consuming, requires very large mosquito samples, and has been found to be less accurate in low transmission areas [11]. Many studies have been reporting the evaluation and validation of polymerase chain reaction (PCR) assays for the detection of malaria in the field. However, costs and technical requirements currently hamper their implementation in endemic areas [12].

Prior to the molecular diagnosis era, serology was used to identify subjects with evidence of current or recent infection, with this method having the potential to detect not only ongoing blood stage infections but also symptomatic or asymptomatic infections [13–15]. In recent years, using serological data to monitor *P. falciparum* and *P. vivax* transmission intensity and exposure has gained recognition in the monitoring of changes in transmission, and in identifying hotspots of transmission and high-risk groups [16–19].

Numerous studies conducted in a variety of epidemiological settings have revealed that the levels and seroprevalence of sporozoite antibodies, especially those against the repetitive epitope of circumsporozoite protein (CSP) of *P. falciparum*, are a predictor of parasite exposure over time and are a good indicator of malaria transmission intensity [20–24]. However, the persistence of antibody responses long after malaria transmission has ceased has been reported in a non-endemic region of Brazil and in a low transmission area in Thailand, and long-lived anti-CSP antibody responses have been detected in the absence of a blood-stage infection [25, 26]. Moreover, antibody responses to sporozoite showed some evidence that host genetics, such as human leukocyte antigen (HLA) class II, do affect antibody responses [27–29].

In naturally exposed individuals in the Brazilian Amazon, the expression of HLA-DR7 has been associated with poor responses against the CSP repetitive region of *P. vivax* (VK210), HLA-DR3, and HLA-DR5, with poor responses against N-terminal region [28–30]. In a phase I trial of *P. falciparum* multiple antigen peptide vaccine, the formulation elicited high levels of parasite-specific antibodies in volunteers expressing DQB1*0603, DRB1*0401, or DRB1*1101 class II molecules [31]. HLA class II molecules may play an important role in antibody response and show huge variability. In humans, this will need to be considered when employing serosurveillance.

In Brazil, a number of cross-sectional studies have aimed to characterize antibody response to CSPs of all three *Plasmodium* species: *P. falciparum*, *P. vivax*, and *P. malariae*. Most of the studies on the antibody responses to CSPs of *P. vivax* and *P. falciparum* point out an association with malaria exposure [24, 32].

In this study, we aimed to test whether seroprevalence to CSPs of *P. falciparum*, *P. vivax*, and *P. malariae* is a good tool for monitoring the exposure to and/or evaluating the burden and distribution of these species in a rural area of Porto Velho, Rondônia state. This endemic area has experienced a decline in *P. falciparum* cases, a progressive increase in *P. vivax* cases, and has had a few reported cases of *P. malariae.* The influence of HLA-DRB1 and HLA-DQB1 allelic products on the antibody responses was also taken into consideration.

## Methods

### Study population and sample collection

This study comprised of cross-sectional surveys being conducted in a rural community of Porto Velho (63^∘^54′13′′W 8^∘^45′43′′S), municipality of Rondônia state, Brazilian Amazon.

In this region, malaria transmission occurs throughout the year with seasonal fluctuations and an increased number of cases in the dry months between April and September. *Anopheles darlingi* is the main vector in all Amazon areas and is the dominant species in Rondônia [33–35]. The numbers of malaria cases in Rondônia were 43 514 in 2010 and 30 412 in 2011 (when the survey was conducted). In both years, *P. vivax* was the predominant species, accounting for more than 88% malaria cases, there were 10.8% of *P. falciparum* cases in 2010 and 7.2% in 2011, and no *P. malariae* cases were reported [36].

Samples and survey data were collected during the dry months of June–September in 2010 and 2011. Study participants were recruited via visits to their houses, which were randomly selected. Those who were diagnosed at the outpatient clinic were also recruited. The enrolment exclusion criteria were as follows: age < 10 years old, pregnancy, breastfeeding, antimalarial drug use, mental disorders, and status as a member of an indigenous population. Informed consent was obtained from all participants and the protocol of this study was reviewed and approved by the Fundação Oswaldo Cruz Ethical Committee (CEP/FIOCRUZ, 492/08).

All individuals answered a questionnaire and were asked if they had any symptoms, such as fever, headaches, chills, myalgia, and/or nausea. After the epidemiological survey was conducted, blood was drawn from each participant by venipuncture and malaria infection was diagnosed by microscopic examination of Giemsa-stained blood smears and PCR using primers specific for genus (*Plasmodium* spp.) and species (*P. falciparum*, *P. vivax*, and *P. malariae*). The amplification protocols have been described previously [37].

Subjects were considered positive for *Plasmodium* infection if they were positive according to the thick blood smear and/or PCR. The parasitological evaluations were performed by examination of 200 fields at 1000-fold magnification under oil immersion. Parasite densities were estimated by counting the number of parasites per microliter of blood (all species and stages) per 200 leukocytes in thick blood films, multiplying this by the number of individual leukocytes, and dividing this by 200. All smear-positive individuals were treated with antimalarial drugs, as according to the protocol of the Brazilian Ministry of Health.

### Peptide synthesis

Synthetic peptides representing the repeated immune-dominant epitope of the CSP were used as an antigen for the detection of immunoglobulin G (IgG) antibodies. For *P. vivax*, all three variant sequences were used: *P. vivax* VK210 DGQPAGDRAAGQPAG-(DRADGQPAG)_2_, *P. vivax* VK247 (ANGAGNQPG)_3_-ANGAGN, and *P. vivax*-like (APGANQEGGAA)_3_. For *P. falciparum* and *P. malariae*, sequences (NANP)_8_ and (GNAA)_2_-GNDA-(GNAA)_4_ were used, respectively.

### Measuring IgG responses

The enzyme-linked immunosorbent assay (ELISA) for the detection of antibodies to the CSP was conducted, as previously described [38]. Briefly, MaxiSorp™ plates (Nunc**,** Carlsbad, CA, USA) were coated with 5μg/ml of the synthetic peptides representing CSP repeats of *P. vivax* (PvCS), *P. falciparum* (PfCS), and *P. malariae* (PmCS), and incubated overnight at 4 °C. Individual plasma samples were assayed at a dilution of 1:100 for one hour at 37 °C. Bound antibodies were detected by IgG-peroxidase conjugated goat anti-human IgG (Sigma-Aldrich, St Louis, MO, USA), followed by O-phenylenediamine dihydrochloride (Sigma-Aldrich, St Louis, MO) and hydrogen peroxide. The optical densities (ODs) were recorded on an ELISA reader (BioTek ELx800™) at 490 nm. On each plate, reactive standard sera and non-reactive standard sera from individuals living in non-endemic areas who have never been exposed to malaria infection were placed. Results were expressed as reactivity index (RI), which was calculated by dividing the mean OD values of tested samples by the mean plus three standard deviations (SDs) of negative controls tested simultaneously. Samples with an RI greater than 1 were considered positive for the CSP peptide tested. In the case of *P. vivax*, positive samples presenting RI > 1 for any of the three CSP repeats representing *P. vivax* CSP genotypes (VK210, VK247, and *P. vivax*-like) were considered.

### HLA genotyping

Genomic DNA was isolated from whole blood with ethylenediaminetetraacetic acid (EDTA) using QIAamp® DNA Blood Midi Kit (QIAGEN, Chatsworth, CA, USA). The concentration and quality of all DNA were analysed using NanoDrop® ND-1000 (Thermo Fisher Scientific, Inc., Waltham, MA, USA). HLA-DRB1 and HLA-DQB1 genes were typed using a Luminex® Multi-analyte profiling system (One Lambda, Inc., Canoga Park, CA, USA). This system uses LABType® SSO One Lambda typing kit (One Lambda, Inc., CA, USA) and is based on the PCR sequence-specific oligonucleotide probes. The highly polymorphic exons 2 of the HLA-DRB1 and HLA-DQB1 genes were amplified using the kit-specific primer pairs. The 5′ ends of the upstream primers were labeled with biotin. Hybridized amplicons were labeled with streptavidin, R-phycoerythrin, and quantified on the Luminex® 100™ flow analyzer (One Lambda, Inc., CA, USA). The hybridized patterns were classified using HLA Visual 2.0 software (One Lambda, CA, USA) according to the manufacturer’s instructions.

### Statistical analysis

Analysis were done using Epi Info™ 2002 (CDC, Atlanta, GA, USA), GraphPad Prism version 7.0 (GraphPad Software, Inc., San Diego, CA, USA), and R version 3.4.0 (Vienna, Austria) [39]. Differences in medians in terms of the study population data were calculated using the non-parametric Mann–Whitney U test. The student’s t-test was used to compare the means of normally distributed data, or normalized transformations were performed on raw data before testing by one-way analysis of variance (ANOVA). Differences in the proportions of the frequencies between variables were evaluated using the chi-square (*χ*^2^) test. Associations between antibody levels and epidemiological data were assessed with Spearman’s rank correlation. Overall associations of antibody responses with the alleles from each HLA-DRB1* and HLA-DQB1* loci were evaluated by comparing the allele frequencies between seronegative (non-responders) and seropositive (responders) subjects using a Fisher’s exact test 2 × 2 contingency table. Statistical significance was set at *P* < 0.05.

## Results

### Characteristics of the study participants

Details about the study population are summarized in Table 1. Three hundred and fifty-seven individuals were enrolled in this study. The majority of individuals included in this study were randomly selected (*n* = 285). Only 72 individuals were recruited from the outpatient clinic, but they lived in the same area as the other participants and had similar exposure to malaria infections.

**Table 1:** Epidemiological characteristics of study participants

		*Malaria diagnosis*			
		Positive	Negative	Total	*P-*value
		*n* = 98	*n* = 259	*n* = 357	
Gender *n* (%)	Female	30 (19.4)	125 (80.6)	155 (43.4)	0.003
	Male	68 (33.7)	134 (66.3)	202 (56.6)	
Age (years)		30.2 ± 11.6	30.5 ± 15.5	30.4 ± 14.5	0.862
Time of residence in endemic area		25.6 ± 11.5	24.2 ± 13.1	24.5 ± 12.7	0.352
Previous malaria infection		7.7 ± 12.4	11.7 ± 15.2	10.6 ± 14.5	0.021
Time since last malaria infection		29.7 ± 69.4	41.6 ± 66.7	38.3 ± 67.5	0.137
Previous *Plasmodium* species					
*P. vivax*		35 (47.3)	71 (32.0)	106 (35.8)	
*P. falciparum*		1 (1.4)	13 (5.9)	14 (4.7)	0.236
*P. vivax***/***P. falciparum*		38 (51.3)	137 (61.6)	175 (59.1)	
Current *Plasmodium* species					
*P. vivax*		–	–	71 (72.5)	
*P. falciparum*		–	–	26 (26.5)	< 0.0001
*P. vivax***/***P. falciparum*		–	–	1 (1.0)	

Gender: *n* (%); Age: mean ± *SD*; Time of residence in endemic area (years): mean ± *SD*; Previous malaria infection: mean ± *SD*; Time since the last malaria infection (months): mean ± *SD*; Previous *Plasmodium* species: *n* (%); Current *Plasmodium* species: *n* (%)

Parasite prevalence determined by microscopy and/or PCR was 27% (71 had P. vivax, 26 had *P. falciparum*, and one had a mixed infection). It is worth noting that all individuals testing positive by microscopy were also positive by PCR, except five individuals who were found positive only by PCR.

The proportion of males to females was not significant, but the infection rate was higher in males. No differences were observed in the mean age, time of residence in endemic area, and time elapsed since the last malaria episode between parasite-positive and parasite-negative individuals. However, individuals who were parasite-negative recalled a higher number of previous malaria episodes than those who were parasite-positive. The studied population recalled, by memory, having had previous episodes with confirmed diagnosis of *P. vivax* or *P. falciparum* malaria, but no history of *P. malariae.*

Malaria-associated symptoms were present in 86.7% of the infected individuals, even though they all reported repeated malaria infections. The most common symptoms reported by the participants at the time of blood collection were fever (66%), headaches (76%), chills (54%), myalgia (57%), and nausea (43%).

### Naturally acquired antibody responses against the CSP

**Fig. 1:**
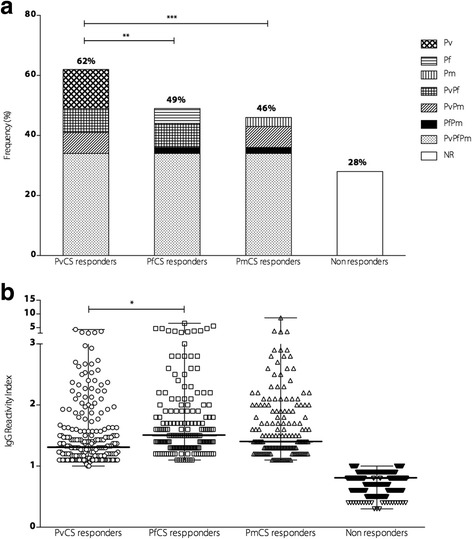
Prevalence and magnitude of antibodies to CSP repeats. **a** Frequency of responders to PvCS, PfCS, and PmCS. Pv represents individuals responding to PvCS peptide only, Pf for PfCS only, Pm for PmCS only, PvPf for both PvCS and PfCS peptides, PvPm for both PvCS and PmCS peptides, PfPm for both PfCS and PmCS peptides, and PvPfPm responders to all three peptides. NR represents non-responders to all three peptides. **b** RI of IgG antibodies against each CS peptide. RI > 1 was considered positive. The bars indicate the median and the range

The prevalence of naturally acquired antibodies to PvCS, PfCS, and PmCS was measured. Of the study participants, 72% presented IgG against at least one of the three peptides. The prevalence of IgG against PvCS (62%) was higher than PfCS (49%, *P* < 0.01) and PmCS (46%, *P* < 0.001)*.* Most of the individuals had antibodies to at least one of the three peptides (72%), 34% had antibodies to all three peptides and 28% were non-responders. Although *P. malariae* was not detected by microscopy or PCR in the study population, the prevalence of antibodies to PmCS (46%) was similar to PfCS (49%) (see Fig. 1a). Levels of antibodies, expressed as the RI, were significantly higher in responders to PfCS than to PvCS (see Fig. 1b). However, the IgG RI of anti-CS antibody was also not associated with time of residence in endemic area (*r* = 0.048 *P* = 0.36 for PvCS; *r* = 0.04, *P* = 0.45 for PfCS, and *r* = 0.058, *P* = 0.27 for PmCS) or number of past malaria infections (*r* = 0.016, *P* = 0.75 for PvCS; *r* = 0.010, *P* = 0.84 for PfCS, and *r* = 0.053, *P* = 0.31 for PmCS). We did not find any association between antibody responses and age (*r* = 0.006, *P* = 0.11 for PvCS; *r* = 0.009, *P* = 0.06 for PfCS, and *r* = 0.004, *P* = 0.21 for PmCS).

### Effect of *P. falciparum* and *P. vivax* infections on IgG positivity and magnitude

**Fig. 2:**
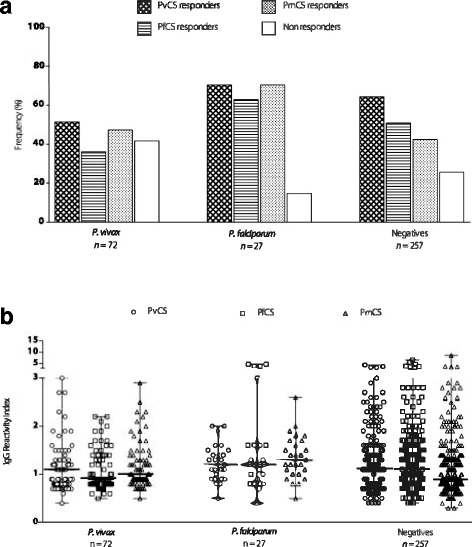
Prevalence and magnitude of antibodies to CSP repeats in parasite-positive and parasite-negative individuals. **a** Frequency of responders to PvCS, PfCS, and PmCS among *P. vivax* and *P. falciparum* parasite-positive individuals and parasite-negative individuals (negatives). **b** IgG RI of antibodies to PvCS, PfCS, and PmCS among *P. vivax* and *P. falciparum* parasite-positive individuals and parasite-negative individuals (negatives). The bars indicate the median and the range

The prevalence and magnitude of specific CSP antibodies in parasite-positive individuals showed that 51.4% of *P. vivax-* and 62.9% of *P. falciparum*-infected individuals had antibodies to the corresponding CSP repeats. In individuals diagnosed with *P. vivax*, the prevalence of antibodies to PvCS was similar to PfCS and PmCS, and 41.7% were non-responders. In individuals diagnosed with *P. falciparum,* the prevalence of antibodies to PfCS was also similar to PvCS and PmCS. However, only 14.8% were non-responders, and the prevalence of antibodies to PfCS and PmCS were significantly higher in individuals with *P. falciparum* when compared to *P. vivax*-infected individuals. Although the majority of the population (72%) was not infected at the time of the survey (*n* = 257), 74.3% of the parasite-negative individuals had antibodies to at least one of the CSPs. Among the parasite-negative individuals, the prevalence of antibodies to PvCS was significantly higher when compared to PfCS (*P* < 0.01) and PmCS (*P* < 0.0001), and 25.7% were non-responders (see Fig. 2a). Comparing the antibody levels between malaria-positive and malaria-negative individuals, the presence of parasites did not have any impact on the IgG RI of responders. Likewise, no significant differences were observed in the magnitude of PvCS antibodies in *P. vivax* parasite-positive individuals and PfCS antibodies in *P. falciparum* parasite-positive individuals. Although individuals who were parasite-negative seemed to have higher antibody levels, no significant differences were observed between parasite-positive and parasite-negative individuals for all three CSPs (see Fig. 2b).

### Antibody responses to CSP according to the time elapsed since the last malaria infection

To investigate the ability of the CSP antibody to determine whether individuals had been infected in the recent past, we compared the prevalence and the RI of anti-CSP antibodies according to the time elapsed since the individuals’ last malaria infection according to their recall in memory.

**Fig. 3:**
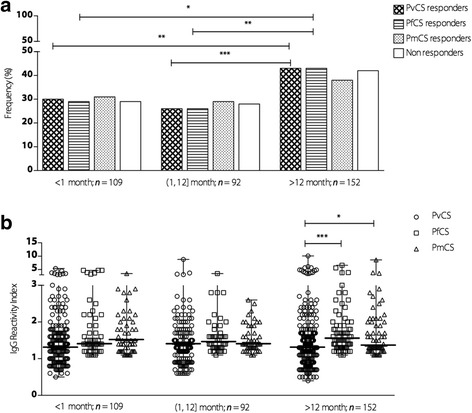
Prevalence and magnitude of antibodies against CSP repeats according to time since the last malaria infection. **a** Frequency of responders to PvCS, PfCS, and PmCS of individuals who recalled the last malaria infection within less than 1 month: < 1 month, more than 1 month and less than 12 months: (1, 12] months, and more than 12 months: > 12 months. **b** IgG RI of antibodies to PvCS, PfCS, and PmCS of individuals who recalled the last malaria infection within less than 1 month: < 1 month, more than 1 month and less than 12 months: (1, 12] months, and more than 12 months: > 12 months. The bars indicate the median and the range

Individuals positive for malaria were assigned as having malaria in the last ≤1 month. Figure 3a shows that the frequency of responders to PvCS, PfCS, and PmCS did not significantly differ between individuals who had been infected more recently (≤1 month and (1, 12] months). In individuals who had the last malaria infection over a year ago (> 12 months), the prevalence rates of antibodies to PvCS and PfCS were higher when compared with individuals more recently infected. The IgG RI to PvCS, PfCS, and PmCS did not differ according to the time since the last malaria infection. It is noteworthy that among individuals who reported their last malaria infection over the past 12 months, IgG levels to PfCS and PmCS were higher when compared to PvCS (see Fig. 3b). However, no association was observed between the IgG RI and time elapsed since the last malaria infection for all the CSP antigens (*r* = 0.009, *P* = 0.85 for PvCS; *r* = 0.015, *P* = 0.78 for PfCS, and *r* = 0.024, *P* = 0.64 for PmCS).

### HLA-DRB1 and HLA-DQB1 allele and antibody responses

Considering that HLA class II molecules play an important role in antibody response and that 28% of individuals did not respond to all three CSP peptides, we evaluated the influence of HLA-DRB1 and HLA-DQB1 alleles on the naturally acquired IgG responses to the CSPs of *P. vivax*, *P. falciparum*, and *P. malariae* in 280 individuals. The frequency of each allele in responders and non-responders to all three CSPs are summarized in Table 2.

**Table 2:** Frequency (f) and number (n) of antibody responders (R) and non-responders (NR) to CS peptide repeats for *P. vivax*, *P. falciparum*, and *P. malariae* according to HLA-DRB1 and HLA-DQB1 genotypes

	PvCS		PfCS		PmCS	
	R	NR	R	NR	R	NR
	f (*n*)	f (*n*)	f (*n*)	f (*n*)	f (*n*)	f (*n*)
HLA-DRB1						
DRB1*01	0.05 (18)	0.15 (30) ***	0.04 (12)	0.13 (36) ***	0.06 (15)	0.11 (33)
DRB1*03	0.06 (23)	0.05 (9)	0.06 (17)	0.05 (15)	0.06 (16)	0.05 (16)
DRB1*04	0.16 (59)	0.15 (29)	0.18 (52)	0.13 (36)	0.14 (35)	0.17 (53)
DRB1*07	0.12 (43)	0.08 (16)	0.13 (36)	0.08 (23)	0.11 (27)	0.10 (32)
DRB1*08	0.07 (27)	0.10 (19)	0.09 (25)	0.08 (21)	0.09 (23)	0.07 (23)
DRB1*09	0.02 (9)	0.03 (6)	0.02 (6)	0.03 (9)	0.02 (6)	0.03 (9)
DRB1*10	0.03 (12)	0.02 (3)	0.01 (4)	0.04 (11)	0.03 (7)	0.03 (8)
DRB1*11	0.11 (40)	0.10 (19)	0.10 (27)	0.12 (32)	0.12 (31)	0.09 (28)
DRB1*12	0.01 (2)	0.03 (5)	0.01 (3)	0.01 (4)	0.01 (3)	0.01 (4)
DRB1*13	0.15 (56)	0.11 (22)	0.15 (41)	0.13 (37)	0.14 (34)	0.14 (44)
DRB1*14	0.04 (16)	0.04 (7)	0.05 (13)	0.04 (10)	0.04 (9)	0.05 (14)
DRB1*15	0.09 (33)	0.10 (19)	0.08 (23)	0.10 (29)	0.10 (26)	0.08 (26)
DRB1*16	0.08 (28)	0.05 (10)	0.08 (23)	0.05 (15)	0.07 (18)	0.06 (20)
HLA-DQB1						
DQB1*02	0.17 (62)	0.12 (24)	0.19 (53)*	0.12 (33)	0.17 (43)	0.14 (43)
DQB1*03	0.40 (145)	0.34 (65)	0.41 (116)	0.34 (94)	0.38 (96)	0.37 (114)
DQB1*04	0.08 (30)	0.08 (16)	0.07 (21)	0.09 (25)	0.06 (16)	0.10 (30)
DQB1*05	0.15 (54)	0.24 (47) **	0.13 (36)	0.23 (65) *	0.17 (42)	0.19 (59)
DQB1*06	0.20 (74)	0.22 (42)	0.20 (55)	0.22 (61)	0.21 (52)	0.21 (64)

The association was verified sing a Fisher’s exact test 2 × 2 contingency table; *P < 0.01, ** *P* < 0.001, *** *P* < 0.0001. Each individual contributed with two HLA allele observations

Common allele groups ranged from 13 at the HLA-DRB1 locus and five at the HLA-DQB1 locus. The predominant HLA-DRB1 allelic groups were HLA-DRB1*04 (16%) and HLA-DRB1*13 (14%), and the most frequent HLA-DQB1 were HLA-DQB1*03 (38%) and HLA-DQB1*06 (21%). Although 72% of the population were positive to at least one of the peptides, the frequency of HLA-DRB1*01 was significantly higher in non-responders to PvCS and PfCS and HLA-DQB1*05 in non-responders to PvCS and PfCS. In contrast, we observed a higher frequency of HLA-DQB1*02 genotype in responders to PfCS peptide. Antibodies to PmCS showed no association with particular HLA-DRB1 and HLA-DQB1 genotypes.

### Evaluation of HLA-DRB1 and HLA-DQB1 haplotypes and antibody responses

Associations attributed to individual DRB1 and DQB1 allele groups were partially reflected by the DRB1~DQB1 haplotypes. These allele groups contributed to 10 common DRB1~DQB1 haplotypes.

**Fig. 4:**
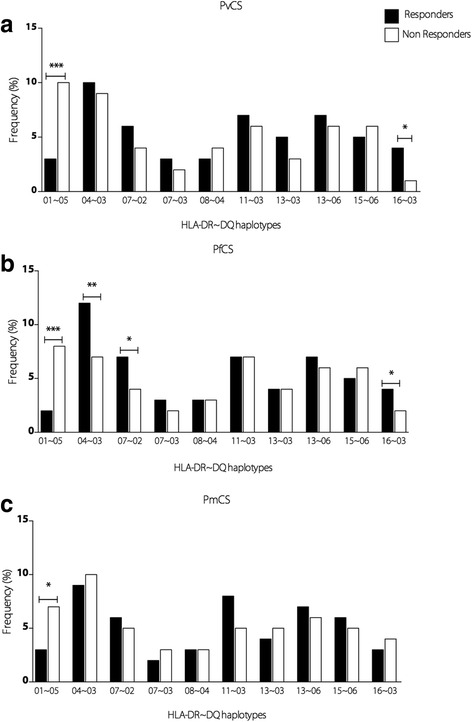
Association of HLA DRB1*DQB1 haplotypes and CS antibody response. Frequency (%) of HLA DRB1*DQB1 haplotypes in IgG responders and non-responders to the CS peptides of **a** PvCS, **b** PfCS, and **c** PmCS. HLA DRB1*DQB1 haplotypes with less than eight occurrences among subjects were not included in the analysis. These haplotypes were considered more frequent

Frequencies of responders and non-responders to each peptide according to the haplotypes are described in Fig. 4. The association between DRB1*01 and DQB1*05 of non-responders was mostly due to DRB1*01~DQB1*05 haplotype for PvCS, PfCS, and PmCS. The positive association of DQB1*02 with responders to PfCS was mainly because of the DRB1*07~DQB1*02 haplotype. Among individuals carrying the haplotypes DRB1*04~DQB1*03, there was a significantly higher frequency of responders to PfCS, and DRB1*16~DQB1*03 haplotype for PvCS and PfCS.

## Discussion

Malaria elimination is on the agenda of the control program in Brazil, and effective monitoring of disease transmission is crucial. From 2007 to 2017, *P. falciparum* has been decreasing (under 11%), *P. vivax* has become the predominant species (88%), and only a few cases of *P. malariae* have been reported in Rondônia state. This epidemiological situation gave us the opportunity to investigate whether seroprevalence to the CSPs of *P. falciparum*, *P. vivax*, and *P. malariae* is a good tool for monitoring the exposure to and/or evaluating the burden and distribution of these species in a rural area of Porto Velho, Rondônia.

Our results show that antibody responses against the CSP repeats of *P. vivax* and *P. falciparum* were higher than those estimated based on blood analysis and/or PCR, and revealed the presence of anti-CSP antibody to *P. malariae*. This is not surprising as a high prevalence of anti-CSP antibody to *P. malariae* has been reported in five states of the Amazon, including Rondônia [32]. Although we did not detect *P. malariae* infection by PCR, this species seems to be more common than suggested by official data based on microscopy. Studies using nested PCR have found significantly more cases of *P. malariae* infection in patients from Rondônia (10%) and Mato Grosso (11.9%) [4, 5, 32]. In addition, case reports documented that *P. malariae* has the ability to persist in a single host for decades as a chronic, low-density, asymptomatic infection, and there is a risk of recurrence decades after initial exposure, even when the infected individuals have left the endemic region [40–42]. Even though *P. malariae* reported cases in Rondonia have been rare, the high prevalence of antibodies to the CSP repeats of *P. malariae* in the absence of detected cases during the surveys could be due to the presence of antibodies cross-reacting with *P. vivax*, *P. falciparum* or with the simian *P. brasilianum* antigens [43]. Indeed, *P. malariae* and *P. brasilianum* share identical CS repeat sequences and do not segregate into distinct types in all genomic markers used so far [44].

Overall, production of anti-CSP antibodies was independent of a blood-stage infection. Current or recent blood infections appear to have little impact on seropositivity and do not seem to boost the anti-CSP response, as prevalence and levels of anti-CSP antibodies were not significantly different between parasite-positive and parasite-negative individuals. This is consistent with other studies that showed the absence of anti-CS antibody responses in individuals with confirmed exposure to *P. falciparum*, *P. vivax* and *P. malariae* [24, 45–48]. However, it also important to note that in the case of *P. vivax*, the presence of blood stage infection does not necessarily indicate a new infection as it may be due to a relapse. It is not known if the presence of hypnozoites/relapses could influence serological profile, especially those against sporozoites.

Numerous studies that evaluated anti-CSP antibody responses in endemic populations showed that an anti-sporozoite humoral immune response was independent of a blood stage infection, but correlates with exposure and increases with age [49–51]. We were unable to establish a correlation between specific anti-CSP responses and age, time of residence in endemic area, number of previous malaria infections, and time elapsed since the last malaria infection, corroborating with previous observations that anti-CSP antibodies do not necessarily correlate with recent exposure [17, 46, 47]. However, seroprevalence indicates ongoing exposure to all three *Plasmodium* species in the area.

As infection and exposure refer to infective bites by a mosquito regardless whether it results in a blood-stage infection, it is difficult to obtain data that reflect individual natural exposure to infective bites. On the other hand, several studies showed that anti-CSP antibody responses change with seasonal or geographical transmission intensity. In low transmission settings, anti-CSP antibodies formed at a slower rate and decayed at a faster rate compared to anti-blood-stage antibodies [21]. In contrast, half-life of antibodies against CSP has been reported as long as several years in a malaria outbreak in Brazil and in Africa, and as short as 27 days in Thailand [25, 52, 53].

Although we were unable to establish a correlation between time since the last malaria infection and specific anti-CSP responses, the prevalence of antibodies to PvCS and PfCS was higher in individuals for whom the time elapsed since the last malaria infection was more than one year ago.

One major limitation of our study is that serological measures were performed from a single cross-sectional survey at the malaria transmission season and changes in prevalences and the persistence of antibodies to the CSP after the end of the transmission was not evaluated. Interestingly, in our study, a proportion of naturally exposed individuals remained unresponsive to all CSP repeats (28%), even after a history of repeated malaria exposures and current *P. falciparum* and *P. vivax* infection. Non-responsiveness to CSP in a proportion of individuals living in endemic areas is a well-documented finding and several studies have reported an association between specific HLA alleles and immune response to malaria antigen [28, 54]. In the current study, HLA-DRB1*01 and HLA-DQB1*05 allelic groups were associated with a lack of antibodies to PvCS and PfCS, and the haplotype DRB1*01~DQB1*05 was also associated with non-responders, including non-responders to *P. malariae*. Interestingly, according to the Brazilian National Registry of Bone Marrow Voluntary Donors, the allelic frequency of DRB1*01 in the Amazon region is 19.73% and in Rondônia, it is 19.79% [55]. In addition, in 99.3% and 98.9% of times (Amazon region and Rondônia, respectively) that both DRB1 and DQB1 were typed, the presence of DRB1*01 was associated with DQB1*05 allele, indicating a total linkage disequilibrium. Considering that the DRB1*01 allele is frequent in the studied population and in the Amazon region, although the ethnicity of the population may impact the frequency of HLA, a great number of individuals could be non-responders. HLA-DRB1*01 was also negatively associated with repetitive regions of *P. vivax* MSP-9 antigen in the Brazilian Amazon, and HLA-DQB1*05 along with HLA-DRB1*13 has been associated with a reduced susceptibility to severe malaria in Gambian children [28, 51, 54].

In contrast, we observed a higher frequency of DRB1*07~DQB1*02 and DRB1*04~DQB1*03 haplotypes in responders to PfCS, and DRB1*16~DQB1*03 haplotype for both PfCS and PvCS. These antibody responses did not seem to be related to exposure as no difference was observed between time of residence, number of previous malaria infection, and time since the last malaria infection, and the presence of these alleles.

## Conclusions

Our results show that in a low transmission setting, naturally acquired antibody responses against the CSP repeats of *P. vivax*, *P. falciparum*, and *P. vivax* measured in a single cross-sectional study may not represent a valuable marker for monitoring recent malaria exposure, especially in an area with a high prevalence of *P. vivax*. Furthermore, HLA class II alleles (HLA-DRB1*01, HLA-DQB1*02, and HLA-DQB1*05) were associated with antibody response for CSP of *P. vivax* and *P. falciparum*, and it will be of interest to consider a HLA analysis when using serosurveillance among genetically diverse populations. In the context of malaria elimination, factors affecting the acquisition and maintenance of antimalarial antibodies are important for the development of serosurveillance tools.

## Additional file

Additional file 1: Multilingual abstracts in the six official working languages of the United Nations. (PDF 666 kb)

## Abbreviations

CS: Circumsporozoite
CSP: Circumsporozoite protein
EIR: Entomological inoculation rate
ELISA: Enzyme-linked immunosorbent assay
HLA: Human leukocyte antigen
IgG: Immunoglobulin G
OD: Optical density
PCR: Polymerase chain reaction
PfCS: Circumsporozoite protein repeats of *P. falciparum*
PmCS: Circumsporozoite protein repeats of *P. malariae*
PvCS: Circumsporozoite protein repeats of *P. vivax*
RDT: Rapid diagnostic test
RI: Reactivity index
*SD*: Standard deviation
