# Supplementary material for: Evaluating seroprevalence to circumsporozoite protein to estimate exposure to three species of Plasmodium in the Brazilian Amazon
Source: Infect Dis Poverty. 2018 May 14;7:46. doi: 10.1186/s40249-018-0428-1 (PMC5950184; doi:10.1186/s40249-018-0428-1)
Supplement: Supplementary file 1 — Multilingual abstracts in the six official working languages of the United Nations. (PDF 666 kb) [file 40249_2018_428_MOESM1_ESM.pdf]

Translation of the abstract into the five official working languages of the United Nations

تقييم الانتشار المصلي *لبروتين* ***CSP*** لتقدير التعرض *للمتصورة النشطة* ***(P. Falciparum)*** *والمتصورة المنجلية* ***(.P vivax)*** *ومتصورة الملاريا* ***(P. malariae)*** في الأمازون البرازيلي.

Virginia Araujo Pereira, Juan Camilo Sánchez-Arcila, Mariana Pinheiro Alves Vasconcelos, Amanda Ribeiro Ferreira, Lorene de Souza Videira, Antonio Teva, Daiana Perce da Silva, Maria Teresa Queiroz Marques, Luzia Helena de Carvalho, Dalma Maria Banic, Luiz Cristóvão de Moraes Sobrinho Pôrto, Joseli Oliveira-Ferreira

ملخص

خلفية: سجّلت البرازيل تقدما ملحوظا في الحدّ من الملاريا كما أن البلد في طريقه للقضاء عليها. ومع ذلك، وللقضاء عليها نهائيا، يحتاج برنامج المكافحة إلى أدوات فعالة من أجل رصد التعرض للملاريا وانتقالها. من خلال هذه الدراسة، نهدف إلى تقييم ما إذا كان الانتشار المصلي لبروتين CSP يعتبر أداة جيدة لرصد التعرض و/أو تقييم عبء وتوزيع أنواع المتصورة في الأمازون البرازيلية.

المناهج: تم إجراء مسوحات مقطعية في منطقة ريفية من بورتو فيلهو، ولاية روندونيا. وقد تم الكشف عن العدوى الطفيلية عن طريق الفحص المجهري وتفاعل البوليميراز المتسلسل. كما تم الكشف عن الأجسام المضادة لتكرار طفيليات بروتين CSP من *المتصورة النشطة*، *والمتصورة المنجلية*، *ومتصورة الملاريا* (PvCS و PfCs و PmCS) باستخدام تقنية فحص الامتصاص المناعي المرتبط بالإنزيم. تمت كتابة مستضد كريات الدم البيضاء البشرية HLA -(DRB1) و DQB1 باستخدام تقنية ®Luminex و ®xMAP.

النتائج: كان معدل سيطرة الغلوبولين المناعي G على ببتيد بروتين CSP *للمتصورة النشطة* (62 ٪) أعلى من ببتيد بروتين CSP *للمتصورة المنجلية* (49 ٪) *ومتصورة الملاريا* (46 ٪). وقد كان لدى معظم الأفراد الذين خضعوا للدراسة أجسام مضادة لواحد على الأقل من الببتيدات الثلاثة (72٪)، 34٪ منهم كان لديهم أجسام مضادة لكل الببتيدات الثلاثة و28٪ لم يظهروا أية استجابة. وعلى الرغم من أن غالبية السكان لم يصابوا في وقت المسح، فإن 74.3٪ من الأفراد المصابين بالطفيل لديهم أجسام مضادة لواحد على الأقل من بروتينات CSP. والأهم من ذلك، كان من بين الأفراد الذين يحملون النمط الفرداني DRB1 * 04 ~ DQB1 * 03، نسبة أعلى بشكل ملحوظ من المستجيبين لأشكال *المتصورة المنجلية*، والنمط الفرداني DRB1 * 16 ~ DQB1 * 03 بالنسبة للمستجيبين لأشكال *المتصورة النشطة والمتصورة المنجلية*. في المقابل، ارتبطت مجموعات الأليلات HLA-DRB1 * 01 و HLA-DQB1 * 05 بنقص الأجسام المضادة لتكرار بروتين CSP لكل من *المتصورة النشطة* و *المتصورة المنجلية*، كما ارتبط النمط الفرداني DRB1 * 01 ~ DQB1 * 05 بغير المستجيبين، بما في ذلك غير المستجيبين *لمتصورة الملاريا*.

الاستنتاجات: تظهر نتائجنا أنه في البيئات التي ينخفض فيها احتمال انتقال العدوى، قد لا تمثل الاستجابة للأجسام المضادة المكتسبة بشكل طبيعي ضد تكرار بروتين CSP *للمتصورة النشطة والمتصورة المنجلية ومتصورة الملاريا*، في دراسة مستعرضة واحدة، مؤشرا مهما لمراقبة الإصابة الحديثة بالملاريا، وخاصة في منطقة ذات معدل انتشار مرتفع للمتصورة النشطة. علاوة على ذلك، تلعب جزيئات HLA من الدرجة الثانية دورًا مهمًا في استجابة الأجسام المضادة وتتطلب المزيد من الدراسة وبعينة أكبر حجما. ومن المهم النظر في تحليل HLA عند استخدام رصد مصلي لمراقبة التعرض للملاريا بين السكان المتنوعين وراثيا.

Translated from English version into Arabic by Noureddine KRIMAT and Hadjela Amina, through

检测环子孢子蛋白进行血清阳性率以评估巴西亚马逊地区恶性疟原虫、间日疟原虫和三日疟原虫的暴露风险

Virginia Araujo Pereira, Juan Camilo Sánchez-Arcila, Mariana Pinheiro Alves Vasconcelos, Amanda Ribeiro Ferreira, Lorene de Souza Videira, Antonio Teva, Daiana Perce da Silva, Maria Teresa Queiroz Marques, Luzia Helena de Carvalho, Dalma Maria Banic, Luiz Cristóvão de Moraes Sobrinho Pôrto and Joseli Oliveira-Ferreira

引言：巴西的疟疾发病率大幅下降，且该国正在逐步推进疟疾消除。然而，为了最终消除疟疾，控制项目需要有效的工具来监测疟疾的暴露与传播。本研究旨在评估环子孢子蛋白(CSP)的血清反应能否作为巴西亚马逊地区监测疟原虫物种的暴露，或评估其负担和分布的有效工具。
方法：我们在朗多尼亚州波尔图的农村地区开展了一项横断面调查；使用显微镜和聚合酶链反应检测原虫感染；运用酶联免疫吸附法检测了间日疟原虫、恶性疟原虫和三日疟(PvCS、PfCS、PmCS)等环子孢子蛋白的抗体；利用荧光光谱技术对人类白细胞抗原(HLA)-DRB1 和 DQB1 基因进行分类。
结果：针对间日疟原虫 CSP 多肽的免疫球蛋白 G 的流行率(62%)高于恶性疟原虫(49%)和三日疟(46%)。大多数被研究的个体至少有三种肽中的一种抗体(72%)，34%的个体同时具有三种肽的抗体，28%为阴性。虽然大多数人在调查时未被检测到感染，但 74.3%的原虫阴性个体至少携带一种 CSPs 抗体。更为重要的是，在携带单倍型 DRB1*04~DQB1*03 的个体中，对 PfCS 应答的频率明显更高，而 DRB1*16~DQB1*03 单倍型对 PvCS 和 PfCS 应答较高。相比之下，HLA-DRB1*01 和 HLA-DQB1*05 等位基因组与缺乏间日疟和恶性疟 CSP 重复的抗体相关，单倍型 DRB1*01~DQB1*05 与无应答相关（包括对三日疟）。
结论：研究结果表明，在低传播区开展的单个横断面研究中，对间日疟原虫、恶性疟原虫和三日疟原虫的 CSP 重复自然获得的抗体反应，目前还不能作为监测疟疾暴露的有效标志。特别是在间日疟高流行区。由于 HLA 第 II 类分子在抗体反应中发挥着重要的作用，需扩大样本量开展进一步研究。此外，HLA 分析在使用血清学方法监测遗传多样性人群中疟疾暴露或将发挥作用。

Translated from English version into Chinese by Xin-Yu Feng, edited by Jin Chen, through

CHINESE CENTER FOR DISEASE CONTROL AND PREVENTION
NATIONAL INSTITUTE OF PARASITIC DISEASES

Évaluation de la séroprévalence de la protéine circumsporozoïte pour estimer l'exposition à *P. falciparum*, *P. vivax* et *P. malariae* dans l'Amazonie brésilienne

Virginia Araujo Pereira, Juan Camilo Sánchez-Arcila, Mariana Pinheiro Alves Vasconcelos, Amanda Ribeiro Ferreira, Lorene de Souza Videira, Antonio Teva, Daiana Perce da Silva, Maria Teresa Queiroz Marques, Luzia Helena de Carvalho, Dalma Maria Banic, Luiz Cristóvão de Moraes

Sobrinho Pôrto, Joseli Oliveira-Ferreira

Résumé

Contexte : Le Brésil a connu une forte baisse du paludisme et s'achemine vers l'élimination de la maladie. Il a cependant besoin, pour y parvenir, d'outils efficaces pour surveiller l'exposition et la transmission du paludisme. Dans cette étude, nous avons cherché à évaluer si la séroprévalence de la protéine circumsporozoïte (CSP) était un bon outil pour surveiller l'exposition et/ou évaluer le fardeau et la distribution des espèces du genre *Plasmodium* dans l'Amazonie brésilienne.

Méthodes : Des enquêtes transversales ont été menées dans une zone rurale de Porto Velho, dans l'état de Rondônia. L'infestation parasitaire a été décelée par microscopie et réaction en chaîne par polymérase. Les anticorps dirigés contre les répétitions de CSP des sporozoïtes de *Plasmodium vivax*, *P. falciparum* et *P. malariae* (PvCS, PfCS et PmCS) ont été détectés en utilisant la technique d'immunodosage enzymatique. Les antigènes des leucocytes humains (HLA) DRB1 et DQB1 ont été typés en utilisant la technologie Luminex® xMAP®.

Résultats : La prévalence de l'immunoglobuline G visant le peptide de CSP de *P. vivax* (62 %) était supérieure à celle visant les peptides de CSP de *P. falciparum* (49 %) et de *P. malariae* (46 %). La plupart des individus étudiés avaient des anticorps dirigés contre au moins un de ces trois peptides (72 %)**,** 34 % contre les trois, et 28 % étaient non-répondeurs. Bien que la majorité de la population n'ait pas été infestée au moment de l'enquête, 74,3 % des individus positifs au parasite avaient des anticorps dirigés contre au moins une des CSP. Fait important, les répondeurs à PfCS étaient significativement plus nombreux parmi les individus porteurs des haplotypes DRB1*04~DQB1*03, et les répondeurs à PvCS et PfCS parmi l'haplotype DRB1*16~DQB1*03. En revanche, les groupes alléliques HLA-DRB1*01 et HLA-DQB1*05 ont été associés à un manque d'anticorps contre les répétitions de CSP de *P. vivax* et de *P. falciparum*, et l'haplotype DRB1*01~DQB1*05 a également été associé à des non-réponses, notamment à *P. malariae*.

Conclusions : Nos résultats montrent que dans les milieux à faible transmission, les réponses d'anticorps acquis naturellement contre les répétitions de CSP de *P. vivax*, *P. falciparum* et *P. malariae* relevées au cours d'une seule étude transversale ne constituent peut-être pas un marqueur utile pour la surveillance de l'exposition récente au paludisme, en particulier dans une zone où la prévalence de *P. vivax* est élevée. En outre, les molécules HLA de classe II jouent un rôle important dans la réponse des anticorps et nécessitent une étude plus approfondie avec une taille d'échantillon plus grande. Il sera intéressant de prendre en compte l'analyse HLA dans le cadre de la sérosurveillance pour surveiller l'exposition au paludisme dans des populations génétiquement diverses.

Translated from English version into French by matoudiallo and Suzanne Assenat, through

TRANSLATORS
WITHOUT BORDERS

Оценка серопревалентности к белку спорозоита для прогнозирования риска заболеваемости малярией, вызванной паразитами видов *P. falciparum*, *P. vivax*и *P.*

***malariae* в бразильской Амазонии**

Вирхиния Араухо Перейра, Хуан Камило Санчес- Арсила, Мариана Пинейро Васконселос, Аманда Рибейро Феррейра, Лорен де Соуза Видейра[1], Антонио Тева, Дайана Ререс да Силва[4], Мария Тереза Кейрош Маркес, Лусия Элена де Карвальо, Далма Мария Банич, Луис Криштован де Мораес Собриньо Порто, Хосели Оливейра-Феррейра

Аннотация

Справочная информация: В Бразилии наблюдается значительное снижение заболеваемости малярией с тенденцией к ликвидации этого заболевания. Однако для окончательной ликвидации заболевания программе по борьбе с малярией требуются эффективные инструменты мониторинга заболеваемости и инфицирования малярией. Мы ставим целью настоящего исследования определить, является ли серопревалентность к белку спорозоита (CSP) адекватным способом мониторинга инфицирования и/или оценки бремени болезни и распространения паразитов *рода плазмодии* в бразильской Амазонии.

Методы: Исследования методом поперечных срезов были проведены в сельской местности Порту-Велью штата Рондония. Заражение паразитической инфекцией определялось с помощью микроскопического исследования и полимеразной цепной реакции. Антитела к дупликациям белка спорозоита *Plasmodium vivax*, *P. falciparum*и *P. malariae* (PvCS, PfCS и PmCS) были обнаружены посредством метода ферментного иммуносорбентного анализа. Лейкоцитарный антиген человека (HLA)-DRB1 и гены DQB1 были типированы с помощью технологии Luminex® xMAP® .

Результаты исследования: Распространенность иммуноглобулина G в пептиде CSP паразита *P. vivax* (62%) оказалась выше, чем в пептиде CSP паразитов *P. falciparum* (49%) и *P. malariae* (46%). У большинства участников исследования обнаружены антитела по крайней мере к одному из трёх пептидов (72%), у 34% имелись антитела ко всем трём пептидам, а у 28% отсутствовала клиническая реакция. Несмотря на то, что большинство населения не было инфицировано в период проведения исследования, 74,3% участников исследования, у которых не было выявлено паразитов, имели антитела по крайней мере к одному из белков CSP. Важно отметить, что среди носителей гаплотипов DRB1*04~DQB1*03 распространенность пациентов с клиническим ответом по PfCS, а среди носителей гаплотипов DRB1*16~DQB1*03 с клиническим ответом по PvCS и PfCS были существенно выше. В то же время наблюдалась связь аллельных групп HLA-DRB1*01 и HLA-DQB1*05 с недостатком антител к дупликациям CSP*P. vivax* и *P. falciparum* , а также связь гаплотипа DRB1*01~DQB1*05 с отсутствием клинического ответа у пациентов, включая отсутствие реакции на *P. malariae*.

Выводы: результаты нашего исследования показывают, что в среде с низкой интенсивность передачи заболевания естественная выработка антител к дупликациям CSP *P. vivax*, *P. falciparum*, а также *P. malariae* в одном исследовании методом поперечных срезов не могут представлять собой значимый маркер для мониторинга недавнего инфицирования малярией, в особенности в зоне с высокой распространенностью паразитов *P. vivax*. Кроме того, молекулы HLA-класса II играют важную роль в выработке антител, которая требует дальнейшего исследования на базе увеличенного объёма выборки. Представляет интерес использование анализа HLA-типирования в рамках эпидемиологического надзора по данным

серологического скрининга для мониторинга заболеваемости малярией среди генетически разнообразных популяций.

Translated from English version into Russian by Galina Dmitrieva and Liudmila Tomanek, through

TRANSLATORS WITHOUT BORDERS

Evaluación de la seroprevalencia de la proteína CSP para estimar la exposición al *P. falciparum*, *P. vivax* y *P. malariae* en la Amazonia brasileña

Virginia Araujo Pereira, Juan Camilo Sánchez-Arcila, Mariana Pinheiro Alves Vasconcelos, Amanda Ribeiro Ferreira, Lorene de Souza Videira[1], Antonio Teva, Daiana Perce da Silva[4], Maria Teresa Queiroz Marques, Luzia Helena de Carvalho, Dalma Maria Banic, Luiz Cristóvão de Moraes Sobrinho Pôrto y Joseli Oliveira-Ferreira

Resumen

Antecedentes: Brasil ha logrado progresos sustanciales en la reducción de la malaria y el país avanza hacia su eliminación. Sin embargo, para su eliminación definitiva el programa de control requiere herramientas efectivas para supervisar la transmisión y exposición a la malaria. Este estudio tiene como finalidad evaluar si la seroprevalencia de la proteína CSP es una buena herramienta para supervisar la exposición a las especies *Plasmodium* y/o para evaluar la carga y distribución de estas especies en la Amazonia brasileña.

Métodos: Se llevaron a cabo estudios transversales en un área rural de Porto Velho, estado de Rondonia. La infección del parásito se detectó mediante microscopia y reacción en cadena de la polimerasa. Mediante la técnica ELISA (ensayo por absorción ligado a enzimas) se detectaron anticuerpos contra las repeticiones CSP de esporozoítos de *Plasmodium vivax*, *P. falciparum* y *P. malariae* (PvCS, PfCS y PmCS). Los genes del antígeno leucocitario humano (HLA)DRB1 y DQB1 se tipificaron usando la tecnología Luminex® xMAP®.

Resultados: La prevalencia de inmunoglobulina G frente al péptido *P. vivax* CSP (62 %) era superior a la del péptido CSP *P. falciparum* (49 %) y *P. malariae* (46 %) La mayoría de los individuos estudiados presentaba anticuerpos por lo menos contra uno de los péptidos (72 %)**,** El 34 % presentaba anticuerpos contra los tres péptidos y el 28 % no mostró ninguna respuesta Aunque la mayoría de la población no estaba infectada en el momento del estudio, el 74,3 % de los individuos con resultados negativos en cuanto a parásitos presentaba por lo menos anticuerpos contra una de las proteínas CSP. Es importante destacar que entre los individuos portadores de los haplotipos DRB1*04 DQB1*03 había una frecuencia significativamente más alta de respondedores a PfCS, y del haplotipo DRB1*16 DQB1*03 para los respondedores a PfCS y PvCS. En cambio, los grupos alélicos HLA-DRB1*01 y HLA-DQB1*05 se estaban asociados con la falta de anticuerpos contra repeticiones CSP de *P. vivax* y *P. falciparum*, en tanto que el haplotipo DRB1*01 DQB1*05 también estaba asociado con los no respondedores, incluidos los no respondedores ante *P. malariae*.

Conclusiones: Nuestros resultados muestran que en entornos de baja transmisión, las respuestas que dan los anticuerpos adquiridos naturalmente contra las repeticiones CSP del *P. vivax*, *P. falciparum* y *P. malariae* presentes en un único estudio transversal no representan un marcador útil para supervisar la exposición reciente a la malaria, especialmente en un área con elevada prevalencia del *P. vivax*. Adicionalmente, las moléculas de antígenos leucocitarios humanos (ALH) de clase II desempeñan una importante función en la respuesta de los anticuerpos y requieren un estudio adicional con muestras más grandes. Será interesante considerar el análisis de antígenos leucocitarios humanos (ALH) cuando se utilice la serovigilancia para supervisar la exposición a la malaria de poblaciones genéticamente diversas.

Translated from English version into Spanish by Luz Miranda-Valencia and María José Galdón, through

TRANSLATORS
WITHOUT BORDERS
